# Appendiceal Diverticulitis Masquerading as Perforated Acute Appendicitis: Diagnostic Pitfalls With a Potential Computed Tomography Radiologic Clue

**DOI:** 10.7759/cureus.91798

**Published:** 2025-09-07

**Authors:** Shivam Gandhi, Gustav A Blomquist, Andrew J Weaver, Krista L Denning, John Diks

**Affiliations:** 1 Gastroenterology, Marshall University Joan C. Edwards School of Medicine, Huntington, USA; 2 Radiology, University of Kentucky College of Medicine, Lexington, USA; 3 Trauma and Surgical Critical Care, Marshall University Joan C. Edwards School of Medicine, Huntington, USA; 4 Pathology, Marshall University Joan C. Edwards School of Medicine, Huntington, USA

**Keywords:** acute appendicitis, appendiceal diverticulitis, ct radiological sign, gas micro-loculation, microperforation

## Abstract

We report the case of a 50-year-old man presenting with acute right lower quadrant pain and leukocytosis. Computed tomography suggested acute appendicitis with possible microperforation. Laparoscopic appendectomy revealed diverticulum-like outpouchings, and histopathology confirmed acute appendiceal diverticulitis with periappendicitis.

Appendiceal diverticulitis is an uncommon but clinically significant entity due to its increased risk of perforation and possible association with neoplasia. The clinical and radiologic findings often mimic acute appendicitis, leading to under-recognition without careful review. In our case, a small gas locule seen on CT, initially interpreted as a microperforation, was retrospectively identified as a diverticulum. This finding highlights the potential diagnostic value of intramural gas micro-loculations and a saccular appendiceal contour on CT, particularly when combined with asymmetric periappendiceal fat stranding and wall thickening.

Awareness of these imaging features may aid in distinguishing appendiceal diverticulitis from routine appendicitis, prompting appropriate surgical management and histopathologic assessment. Recognition of this condition is important for timely treatment and for guiding follow-up, given the associated risks.

## Introduction

Appendiceal diverticulosis, first documented by Kelynack in 1893, is a rare pathological condition with clinical importance [1]. Its incidence has been reported in only 0.004% to 2.1% of appendectomy specimens [1]. In a recent retrospective review of 1,586 appendectomies, diverticular disease of the appendix was identified in just 0.63% of cases, underscoring its uncommon occurrence [2]. This condition is more frequently seen in males over the age of 38 and has been associated with disorders including cystic fibrosis and Hirschsprung’s disease [1,3]. 

The etiology of appendiceal diverticulosis is not fully understood but is thought to be acquired in most cases, with pseudodiverticula developing via herniation of the mucosa and submucosa through the muscularis layer of the appendix [1]. Proposed mechanisms include increased intraluminal pressure-often due to luminal obstruction by a fecalith, stricture, or tumor-and repeated low-grade inflammation or infection, which can weaken the appendiceal wall [1]. Identified risk factors include male sex, age above 30 years [1], Hirschsprung’s disease [4], and cystic fibrosis [5]. Some studies have also reported a significant association between appendiceal diverticulosis and neoplasms, with underlying tumors such as carcinoid or mucinous adenoma found in up to 48% of cases in one series [1]. 

Clinically, appendiceal diverticulitis - inflammation of an appendiceal diverticulum - typically presents with symptoms indistinguishable from acute appendicitis, including right lower quadrant abdominal pain, nausea, and localized tenderness [1,2]. This similarity often complicates preoperative diagnosis, so most cases are only definitively diagnosed after surgical intervention and histopathological examination [2]. Despite its rarity, appendiceal diverticulitis carries significant clinical importance due to a markedly higher risk of perforation - estimated to be at least fourfold greater than that of typical acute appendicitis - which can lead to increased morbidity if not promptly recognized and managed [1,2]. Furthermore, both appendiceal diverticulosis and diverticulitis have been associated with an elevated risk of underlying neoplasms, including neuroendocrine (carcinoid) tumors and mucinous adenomas [2,6]. 

These risks underscore the need for careful pathological evaluation and vigilant postoperative follow-up when diverticular disease is identified [2,6]. While appendectomy remains the standard treatment for both acute appendicitis and appendiceal diverticulitis, some authors recommend prophylactic removal if a diverticulum is found incidentally, to mitigate the potential for future complications [1]. 

## Case presentation

A 50-year-old man presented to the emergency department with acute abdominal pain that initially localized to the periumbilical region and gradually migrated to the right lower quadrant. He reported mild nausea but denied vomiting, and his bowel movements were normal. He had no significant past medical history. On examination, the patient appeared uncomfortable but was hemodynamically stable. Vital signs were within normal limits, except for a temperature of 37.8°C (100°F). Physical examination revealed abdominal tenderness in the right lower quadrant, left lower quadrant, and periumbilical regions. Both the obturator and psoas signs were positive, which indicates irritation or inflammation of the psoas or obturator muscle caused by an inflamed retrocecal or pelvic appendix.

The laboratory evaluation demonstrated leukocytosis (11.93 k/µL) with neutrophilia (9.21 k/µL, 77.3%), consistent with an acute inflammatory process. Urinalysis was largely unremarkable except for positive ketones, which may reflect reduced oral intake. Chemistry studies showed a mildly elevated AST and borderline creatinine with preserved eGFR, while other electrolytes, renal, and hepatic parameters were within normal limits. These findings supported the clinical impression of acute appendiceal pathology without evidence of systemic metabolic derangement (Table 1). 

**Table 1:** Laboratory results with reference ranges and Interpretation

Test	Result	Reference Range	Interpretation
Blood Gases			
Lactic Acid	0.7 mmol/L	0.5–2.0 mmol/L	Normal
Hematology			
WBC	11.93 k/µL	4.0–10.5 k/µL	Elevated (leukocytosis)
RBC	4.8 m/µL	4.5–5.9 m/µL	Normal
Hemoglobin	15.8 g/dL	13.5–17.5 g/dL	Normal
Hematocrit	45.9%	41–53%	Normal
Platelets	183 k/µL	150–450 k/µL	Normal
MCV	95.6 fL	80–100 fL	Normal
MCH	32.9 pg	27–34 pg	Normal
MCHC	34.4 g/dL	32–36 g/dL	Normal
RDW-CV	12.8%	11.5–14.5%	Normal
RDW-SD	45.1 fL	35–45 fL	Mildly elevated
MPV	9.8 fL	7.5–11.5 fL	Normal
Neutrophils %	77.3%	40–70%	Elevated
Lymphocytes %	16.4%	20–45%	Low
Monocytes %	5.2%	2–8%	Normal
Eosinophils %	0.3%	0–4%	Normal
Basophils %	0.5%	0–1%	Normal
Neutrophils Absolute	9.21 k/µL	1.8–7.5 k/µL	Elevated
Lymphocytes Absolute	1.96 k/µL	1.0–4.0 k/µL	Normal
Monocytes Absolute	0.62 k/µL	0.2–0.8 k/µL	Normal
Eosinophils Absolute	0.04 k/µL	0.0–0.5 k/µL	Normal
Basophils Absolute	0.06 k/µL	0.0–0.2 k/µL	Normal
Chemistry			
Sodium	140 mEq/L	135–145 mEq/L	Normal
Potassium	4.5 mEq/L	3.5–5.0 mEq/L	Normal
Chloride	106 mEq/L	98–107 mEq/L	Normal
CO₂ (Bicarbonate)	28 mEq/L	22–29 mEq/L	Normal
Anion Gap	6	6–12	Normal
BUN	13 mg/dL	7–20 mg/dL	Normal
Creatinine	1.3 mg/dL	0.7–1.3 mg/dL	Upper normal
eGFR (CKD-EPI)	67 mL/min/1.73 m²	>60	Mildly decreased
Glucose	92 mg/dL	70–99 mg/dL	Normal
Calcium	9.9 mg/dL	8.5–10.5 mg/dL	Normal
Albumin	4.7 g/dL	3.5–5.0 g/dL	Normal
Total Protein	7.1 g/dL	6.0–8.3 g/dL	Normal
Alkaline Phosphatase	77 U/L	40–129 U/L	Normal
AST	41 U/L	10–40 U/L	Slightly elevated
ALT	45 U/L	7–56 U/L	Normal
Total Bilirubin	0.7 mg/dL	0.2–1.2 mg/dL	Normal
Lipase	11 U/L	10–60 U/L	Normal
Urinalysis			
Appearance	Clear	Clear	Normal
Color	Yellow	Yellow	Normal
Specific Gravity	1.026	1.005–1.030	Normal
pH	5	4.5–8.0	Normal
Bilirubin	Negative	Negative	Normal
Urobilinogen	Negative	0–1 EU/dL	Normal
Occult Blood	Negative	Negative	Normal
Glucose	Negative	Negative	Normal
Ketones	20 (positive)	Negative	Abnormal
Protein	Negative	Negative	Normal
Nitrite	Negative	Negative	Normal
Leukocyte Esterase	Negative	Negative	Normal

**Figure 1:**
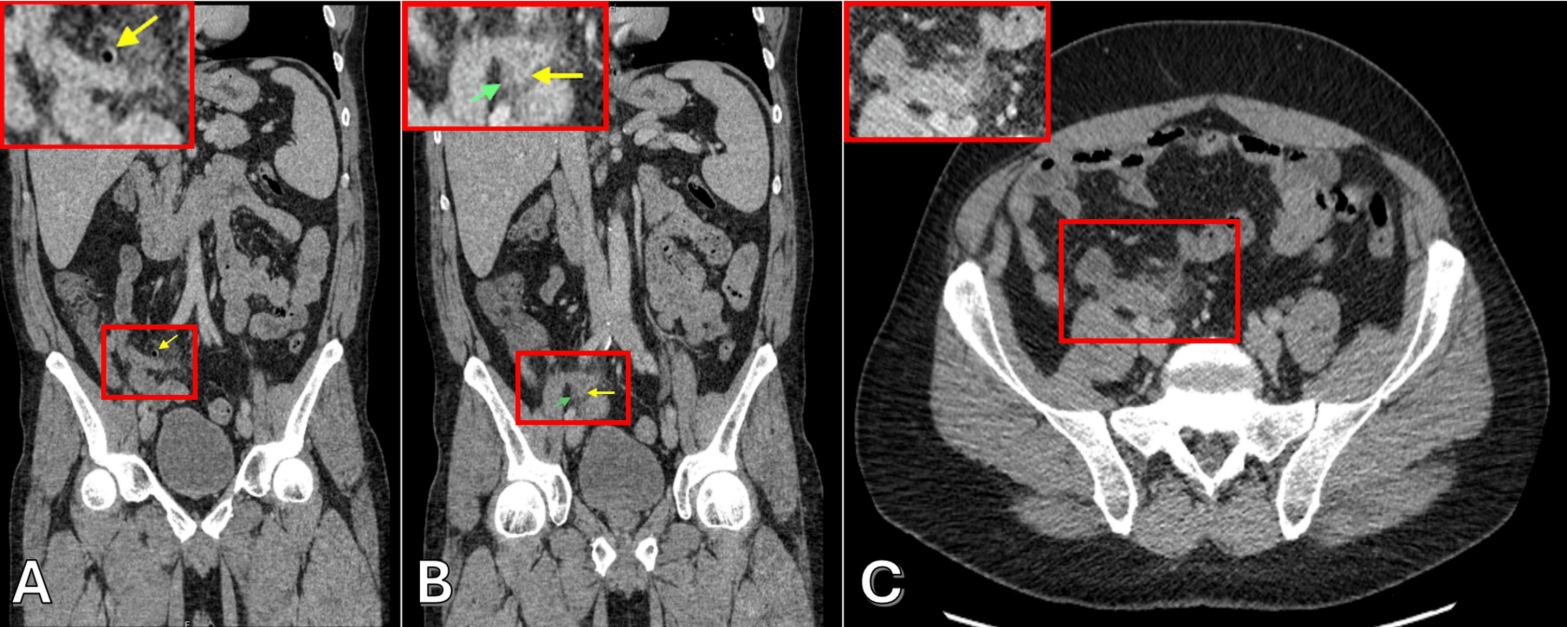
CT (computed tomography) scan images of appendiceal diverticulosis and diverticulitis. (A) Coronal view demonstrates the appendix (red box, with magnified insert) with gas micro-loculation (yellow arrow), a diverticular outpouching containing gas without inflammation. (B) Coronal image demonstrating a saccular appendix (red box, with magnified insert) with periappendiceal inflammatory changes (green arrow) and a fluid-filled diverticulum (yellow arrow), consistent with diverticulitis. (C) Axial image demonstrating a 1.4 cm thickened distal appendix with asymmetric periappendiceal fat stranding (red box, with magnified insert), further supporting the diagnosis of diverticulitis.

Computed tomography (CT) imaging demonstrated acute appendicitis, evidenced by periappendiceal fat stranding and a small locule of extraluminal air, consistent with at least a microperforation (Figure 1) but without evidence of abscess formation. Additional findings included splenomegaly measuring 14.6 cm, a retroaortic left renal vein, and a distended urinary bladder with wall thickening but no signs of inflammation. All other evaluated structures - including the lung bases, liver, gallbladder, pancreas, adrenals, kidneys, bowel (excluding the appendix), abdominal aorta, lymph nodes, body wall, and bones - were within normal limits, and no free fluid was observed in the abdomen or pelvis. 

**Figure 2:**
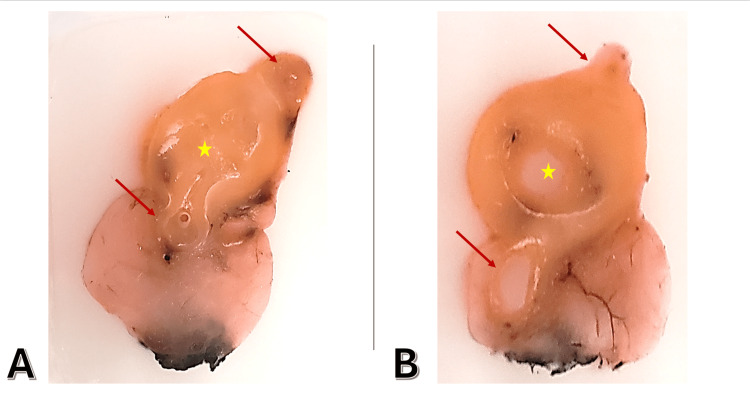
Gross pathology of the appendix shows diverticulosis and diverticulitis. (A and B) Longitudinal sections demonstrate two diverticula outpouching into the peri-appendiceal fat (red arrows) adjacent to the thickened wall and the main appendiceal lumen (yellow star).

The patient underwent emergent laparoscopic appendectomy. Gross examination of the surgical specimen revealed a 6.5 cm intact appendix with multiple areas of gray-tan fibropurulent material adherent to the tip. Sectioning of the appendix demonstrated luminal stenosis by gray-tan, semi-solid tissue proximally and multiple diverticulum-like outpouchings in the distal third containing semi-translucent viscous fluid (Figure 2).

**Figure 3:**
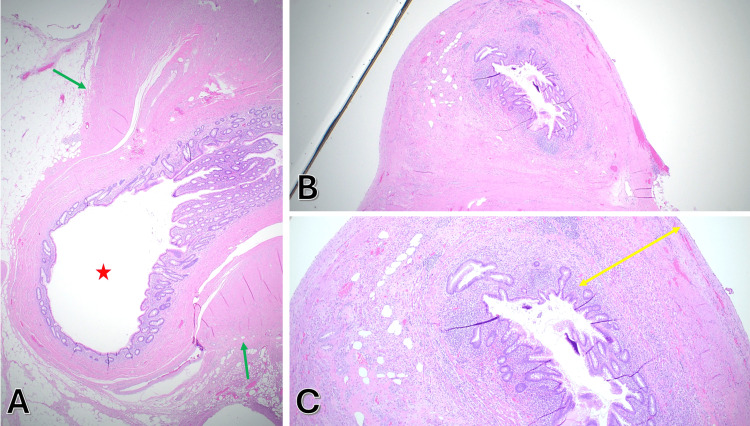
Histopathology of appendiceal diverticulosis & diverticulitis, Hematoxcylin and Eosin (H&E stain). (A) Low-power view (x200) of a diverticulum (red star) with mucosa, submucosa, and attenuated muscularis propria (green arrows). (B) Cross-section (x200) showing acute inflammation involving the diverticular wall. (C) Higher magnification (x400) of the same area demonstrating transmural inflammatory infiltrates extending into periappendiceal tissue (double-headed yellow arrow).

While these findings were suggestive of diverticular disease, it is important to note that gross appearance and clinical presentation alone cannot distinguish diverticulitis from other pathologies, including neoplasms, which may present similarly. Definitive diagnosis was made through histopathological examination, confirming appendiceal diverticulitis with acute periappendicitis. The examination showed acute transmural inflammation in some diverticula while sparing the appendiceal wall, supporting the diagnosis of appendiceal diverticulosis with diverticulitis rather than simple acute appendicitis (Figure 3).

## Discussion

Patients with acute appendicitis typically present with periumbilical pain migrating to the right lower quadrant, tenderness at McBurney’s point, fever, nausea, and leukocytosis. These clinical features of acute appendicitis often mirror those of appendiceal diverticulitis, creating difficulty in distinguishing the two conditions preoperatively [1,2]. Acute appendicitis generally affects a slightly younger demographic, with most patients being 10-40 years old, compared to diverticulitis of the appendix, which often occurs in patients around 38 years of age [3]. Radiologically, uncomplicated appendicitis on CT appears as an enlarged (>6 mm) tubular appendix with wall thickening and periappendiceal fat stranding. An appendicolith may be seen, and perforation is indicated by extraluminal air or abscess formation [7]. Histopathologically, acute appendicitis is characterized by neutrophilic infiltration of the appendiceal wall that starts in the mucosa and extends transmurally (to the muscularis and serosa) in advanced cases. If perforation occurred, frank necrosis and inflammatory debris with periappendiceal abscess may be seen [7]. Surgical removal via appendectomy remains the standard treatment for most acute appendiceal conditions, including both appendicitis and appendiceal diverticulitis [7]. 

Although appendiceal diverticulitis closely mimics acute appendicitis clinically and radiologically, CT typically shows appendiceal wall thickening and periappendiceal fat stranding. A distinguishing feature is the presence of diverticular outpouchings - small, round projections from the appendiceal lumen - that are often subtle and usually identified only retrospectively [1,2]. In our case, a tiny gas locule adjacent to the appendix was seen on CT and interpreted as a microperforation (free extraluminal air); retrospectively, this likely represented an air-filled diverticulum rather than a true perforation. This highlights a radiologic clue for appendiceal diverticulitis: a round gas or fluid-filled outpouching on the appendix in the absence of a clear perforation may indicate a diverticulum. However, preoperative imaging rarely provides a definitive diagnosis; one large review found that only ~4% of appendiceal diverticulitis cases were identified prospectively by CT, with the vast majority diagnosed by postoperative pathology [2]. Experienced radiologists have noted that an inflamed diverticulum can appear on CT as a small cystic or air-filled structure protruding from the appendix with an enhancing wall, which, if recognized, can help differentiate diverticulitis from simple appendicitis [8]. Tissue analysis reveals appendiceal diverticulitis is diagnosed by identifying one or more diverticula - typically acquired pseudodiverticula consisting of mucosa and submucosa herniating through defects in the muscular layer - accompanied by acute inflammation in the diverticulum and often in the adjacent appendiceal wall (peri-diverticulitis). Neutrophil-rich exudate and tissue destruction centered on the diverticulum confirm the diagnosis [9]. In our case, multiple diverticula were identified microscopically with surrounding acute inflammation, consistent with diverticulitis. Notably, if appendiceal diverticulosis (not diverticulitis) is discovered incidentally, removal is still recommended due to its high risk of subsequent perforation [1,2]. 

While appendiceal diverticulitis shares many features with acute appendicitis, it is also important to distinguish it from colonic diverticulosis and diverticulitis, which represents a separate entity with its own clinical implications. Colonic diverticulosis, a common condition in older adults (affecting over 50% of people by 60 years of age), is distinct from appendiceal diverticulosis, which is extremely rare (identified in less than 2% of appendectomy specimens) [1, 10]. Although both conditions involve the formation of diverticular outpouching, there is no well-documented association between colonic and appendiceal diverticulosis, and their co-occurrence is considered coincidental and has been reported only in isolated cases [11]. In our case, appendiceal diverticulitis occurred independently, with no evidence of colonic diverticulosis, appendiceal tumor, or luminal obstruction on imaging or pathology, suggesting a primary diverticular inflammation rather than a secondary process. 

In addition to inflammatory conditions, neoplastic processes of the appendix must also be considered, as they can closely mimic both appendicitis and diverticulitis in their clinical presentation. By patient presentation, appendiceal tumors can mimic the presentation of appendiceal diverticulitis, often manifesting with right lower quadrant pain and symptoms suggestive of acute appendicitis [12]. Imaging typically demonstrates, by contrast, appendiceal tumors often manifest on imaging as focal masses or mucoceles rather than simple wall thickening, helping distinguish neoplasms from inflammatory conditions [12]. Pathologic evaluation shows that appendiceal neoplasms, including neuroendocrine (carcinoid) tumors or mucinous neoplasms, are identified by morphological analysis of neoplastic cells forming masses or exhibiting infiltrative patterns. Carcinoids, for example, show nests or cords of uniform cells with neuroendocrine features, often in the distal appendix, whereas mucinous tumors produce mucin-filled glands or cysts [12]. 

Another important differential diagnosis, particularly in women of reproductive age, is appendiceal endometriosis, which can also present with symptoms and imaging findings similar to acute appendicitis and diverticulitis. By symptom profile, appendiceal endometriosis can resemble appendiceal diverticulitis, presenting with right lower quadrant pain and symptoms similar to acute appendicitis, particularly in women of reproductive age [13]. CT/MRI findings suggest appendiceal endometriosis may appear as a nodular or cystic lesion causing segmental thickening of the appendix, especially in women with known endometriosis [13]. Histology reveals appendiceal endometriosis is diagnosed by the presence of endometrial glands and stroma within the appendix wall [13]. This can be an incidental finding; in cases where it causes symptoms, there may also be surrounding inflammation or even obstruction of the lumen [14]. 

Finally, parasitic infections of the appendix, though rare, represent another potential mimic of inflammatory appendiceal conditions and should be considered in the differential diagnosis. In clinical presentation, parasitic appendicitis can mimic appendiceal diverticulitis, presenting with right lower quadrant pain and symptoms similar to acute appendicitis [15]. Imaging is typically not diagnostic for parasitic appendicitis-an appendix harboring parasites usually shows either a normal appendix or nonspecific inflammatory changes; no specific radiologic sign reliably indicates a parasitic infection preoperatively [15]. Histologic sections reveal parasitic appendicitis is confirmed when sections of the appendix reveal parasites or eggs within the lumen or embedded in the wall. The most frequent offender, Enterobius vermicularis (pinworm), appears as a slender nematode in cross-section in the lumen or crypts, often with mild eosinophilic infiltrates in the tissue [15]. Other parasites (e.g., *Ascaris lumbricoides*, *Schistosoma*, *Strongyloides*) can also be identified in histology on rare occasions [15]. 

A comparative analysis of the principal clinical, radiologic, histopathologic, and management characteristics distinguishing acute appendicitis, appendiceal diverticulitis, and relevant differential diagnoses is presented in Table 2. Incorporating metrics for perforation risk and preoperative diagnostic accuracy elucidates significant prognostic differences and reinforces the necessity of precise entity differentiation for optimal therapeutic decision-making and timing.

**Table 2:** Key Differentiating Features of Acute Appendicitis and Related Conditions Data adapted from multiple sources, including Di Saverio et al., 2020 [7]; Sohn et al., 2013 [16]; Yamana et al., 2012 [17]; Sell et al., 2021 [18]; Rutledge et al., 1992 [5]; Carr et al., 2016 [12]; Mittal et al., 1981 [14]; and Gümüş et al., 2021 [19]. RLQ: right lower quadrant; LLQ: left lower quadrant

Condition	Age Range	Common Symptoms	CT Findings	Histopathology	Preoperative Diagnosis Rate	Perforation Risk	Standard Treatment
Acute appendicitis [7]	10–40 yrs	RLQ pain after periumbilical onset, fever, nausea, leukocytosis	Enlarged (>6 mm) appendix, wall thickening, fat stranding, ± appendicolith, ± perforation	Neutrophilic infiltration, mucosa → transmural	High	16-40% [7]	Appendectomy
Appendiceal diverticulitis [16,17]	Mean ~38 yrs	RLQ pain, GI symptoms; mimics appendicitis	Similar to appendicitis + subtle gas/fluid-filled diverticulum with enhancing wall	Diverticulum with mucosa/submucosa herniation, inflammation centered on diverticulum	Low (~2%)	33-66% [15,16]	Appendectomy (also for incidental diverticulosis)
Colonic diverticulitis [18]	>50 yrs	LLQ pain, fever, bowel habit changes	Colonic diverticula, segmental wall thickening, fat stranding, ± abscess	Diverticula through muscularis with inflammation	High	~1% [17]	Antibiotics ± surgery
Appendiceal neoplasms [5,12]	Any age	RLQ pain; can mimic appendicitis/diverticulitis	Focal mass, mucocele, cystic lesion	Neoplastic cells (e.g., carcinoid nests, mucinous glands)	Variable	Variable	Resection per tumor type
Appendiceal endometriosis [14]	Women of reproductive age	RLQ pain, may be cyclical, linked to menses	Nodular or cystic lesion, segmental thickening	Endometrial glands/stroma in appendix wall	Low	Rare (~0.1%) [18]	Appendectomy if symptomatic/incidental
Parasitic appendicitis [19]	Any age (often children for *Enterobius*)	RLQ pain, mimics appendicitis	Usually normal or nonspecific	Parasites/eggs in lumen or wall, mild eosinophilia	Very low	Low [19]	Appendectomy

## Conclusions

Appendiceal diverticulitis is an uncommon but clinically important diagnosis due to its elevated risk of perforation and possible association with neoplasia. Its clinical and imaging features closely overlap with acute appendicitis, making histopathologic confirmation essential. In our case, a tiny gas locule on CT was initially interpreted as micro-perforation but was later recognized as a diverticulum on pathology. We propose that gas micro-loculations within the appendiceal wall and a saccular appearance of the appendix on CT may indicate appendiceal diverticulosis. When accompanied by asymmetric periappendiceal fat stranding and thickened appendiceal wall, these findings are most consistent with appendiceal diverticulitis, aiding in future diagnostic differentiation. Awareness of this finding, along with the condition’s risks, can guide timely surgical management, appropriate postoperative follow-up, and improved patient outcomes. 

## References

[1] Lesi OK, Probert S, Iqbal MR (2022). Diverticulitis and diverticulosis of the appendix: a case series. Cureus.

[2] Ergenç M, Uprak TK (2022). Appendiceal diverticulitis presenting as acute appendicitis and diagnosed after appendectomy. Cureus.

[3] Friedlich M, Malik N, Lecompte M, Ayroud Y (2004). Diverticulitis of the appendix. Can J Surg.

[4] Dupre MP, Jadavji I, Matshes E, Urbanski SJ (2008). Diverticular disease of the vermiform appendix: a diagnostic clue to underlying appendiceal neoplasm. Hum Pathol.

[5] Rutledge RH, Alexander JW (1992). Primary appendiceal malignancies: rare but important. Surgery.

[6] Elkhawaga M, Mundasad B, Hampton J, Alam AS (2022). Appendiceal diverticulitis presenting as acute appendicitis: a case report. Cureus.

[7] Di Saverio S, Podda M, De Simone B (2020). Diagnosis and treatment of acute appendicitis: 2020 update of the WSES Jerusalem guidelines. World J Emerg Surg.

[8] Vidović S, Čekić N, Šuvak I, Ugljarević M, Pogorelić Z (2025). Acute appendicitis or appendiceal diverticulitis? A case report and systematic literature review. Clin Pract.

[9] Chen JL, Kalidindi V, Mayor-Jerez J, Sadler TJ, Bell DJ (2025). Appendiceal diverticulitis: a rare pathology disguised as acute appendicitis. BJR Case Rep.

[10] Hawkins AT, Wise PE, Chan T (2020). Diverticulitis: An update from the age old paradigm. Curr Probl Surg.

[11] Albeeshi MZ, Alwanyan AA, Salim AA, Albabtain IT (2019). Appendiceal diverticulitis presenting as acute appendicitis diagnosed postoperatively. J Surg Case Rep.

[12] Carr NJ, Cecil TD, Mohamed F (2016). A consensus for classification and pathologic reporting of pseudomyxoma peritonei and associated appendiceal neoplasia: the results of the Peritoneal surface Oncology Group International (PSOGI) modified Delphi process. Am J Surg Pathol.

[13] Allahqoli L, Mazidimoradi A, Momenimovahed Z, Günther V, Ackermann J, Salehiniya H, Alkatout I (2023). Appendiceal endometriosis: A comprehensive review of the literature. Diagnostics (Basel).

[14] Mittal VK, Choudhury SP, Cortez JA (1981). Endometriosis of the appendix presenting as acute appendicitis. Am J Surg.

[15] Agholi M, Esfandiari F, Heidarian HR, Khajeh F, Sharafi Z, Masoudi E, Rayani M (2023). The histopathological findings in appendectomy specimens in an Iranian population. Galen Med J.

[16] Sohn TJ, Chang YS, Kang JH (2012). Clinical characteristics of acute appendiceal diverticulitis. J Korean Surg Soc.

[17] Yamana I, Kawamoto S, Inada K, Nagao S, Yoshida T, Yamashita Y (2012). Clinical characteristics of 12 cases of appendiceal diverticulitis: a comparison with 378 cases of acute appendicitis. Surg Today.

[18] Sell NM, Stafford CE, Goldstone RN (2021). Delay to intervention for complicated diverticulitis is associated with higher inpatient mortality. J Gastrointest Surg.

[19] Gümüş S, Söğütçü N (2021). Parasitic appendicitis in 14.797 cases: a retrospective cohort study. Turkiye Parazitol Derg.

